# Implementation and adaptation of the Re-Engineered Discharge (RED) in five California hospitals: a qualitative research study

**DOI:** 10.1186/s12913-017-2242-z

**Published:** 2017-04-19

**Authors:** S. E. Mitchell, G. M. Weigel, V. Laurens, J. Martin, B. W. Jack

**Affiliations:** Department of Family Medicine, Boston Medical Center, 751 Albany St Dowling building 5th floor south, Boston, MA 02118 USA

**Keywords:** Discharge, Readmissions, Implementation, Adaptation, Fidelity, Leadership, Hospital culture, Sustainability

## Abstract

**Background:**

Project Re-Engineered Discharge (RED) is an evidence-based strategy to reduce readmissions disseminated and adapted by various health systems across the country. To date, little is known about how adapting Project RED from its original protocol impacts RED implementation and/or sustainability. The goal of this study was to identify and characterize contextual factors influencing how five California hospitals adapted and implemented RED and the subsequent impact on RED program sustainability.

**Methods:**

Participant observation and key informant and focus group interviews with 64 individuals at five California hospitals implementing RED in 2012 and 2013 were conducted. These involved hospital leadership, personnel responsible for Project RED implementation, hospital staff, and clinicians. Interview transcripts were coded and analyzed using a modified grounded theory approach and constant comparative analysis.

**Results:**

Both internal and external contextual factors were identified that influenced hospitals’ decisions on RED adaptation and implementation. These also impacted RED sustainability. External factors included: impending federal penalties for hospitals with high readmission rates targeting specific diagnoses, and access to external funding and technical support to help hospitals implement RED. Internal or organizational level contextual factors included: committed leadership prioritizing Project RED; RED adaptations; depth, accountability and influence of the implementation team; sustainability planning; and hospital culture. Only three of the five hospitals continued Project RED beyond the implementation period.

**Conclusions:**

The sustainability of RED in participating hospitals was only possible when hospitals approached RED implementation as a transformational process rather than a patient safety project, maintained a high level of fidelity to the RED protocol, and had leadership and an implementation team who embraced change and failure in the pursuit of better patient care and outcomes. Hospitals who were unsuccessful in implementing a sustainable RED process lacked all or most of these components in their approach.

**Electronic supplementary material:**

The online version of this article (doi:10.1186/s12913-017-2242-z) contains supplementary material, which is available to authorized users.

## Background

The Federal government’s decision to issue penalties to US hospitals for excess readmission rates, spurred hospital leaders to place greater emphasis on improving the hospital discharge process and reducing readmissions [1]. As a result, a number of notable care transition interventions emerged from the patient safety research arena –Project Re-Engineered Discharge (RED), Project Boost and the Coleman Care Transition Program among others —all reporting marked reductions in readmission rates in clinical trials [2–5]. At the same time, Medicare’s Quality Improvement Organizations program (QIO) and the Community-Based Care Transitions Program (CCTP), made the recommendation to implement care transition programs to improve the discharge process and reduce readmissions. This convergence of federal policy, financial penalties, QIO and CCTP recommendations and publication of encouraging research prompted the swift and often not well thought out adoption of the newly developed care transition interventions, with little understanding of the contextual factors that influence the success or failure of such interventions in a new environment.

One successful care transition model is Project RED, created by researchers at Boston Medical Center to reduce readmissions. RED prioritizes a comprehensive discharge process that properly prepares patients for the transition from hospital to home. Project RED is comprised of twelve components focused on key aspects of the discharge process, including patient education, medication reconciliation, communication with and among health professionals, and follow-up care. In a randomized control trial with 749 English-speaking hospitalized adults in 2008, Project RED demonstrated a 30% reduction in hospital utilization post-discharge as compared to individuals receiving usual care, with an average total cost savings of $412 per patient receiving RED [3, 6, 7]. Since completion of the RED clinical trial, hospitals and skilled nursing facilities across the country have implemented RED with varying degrees of success. Some hospitals were able to integrate RED fully into their standard operations, while some were not able to get the program off the ground, and others fell somewhere in between. All hospitals encountered myriad challenges to the implementation of RED, stemming from various contextual factors (e.g. policy, external funding, organizational structure and culture among others) [8–10].

To better understand the real world dissemination of Project RED, our team studied the experiences of hospitals implementing Project RED. In 2015, we reported our initial results on the implementation of RED completed at ten US hospitals. This work highlighted facilitators and barriers to implementing the RED toolkit and that hospital implementation teams frequently made adaptations to the RED protocol. However, our study did not identify the contextual factors that influenced a hospital’s decisions on how to adapt RED and the implications of these decisions on sustainability. We therefore conducted a second study of the implementation experiences of five California hospitals that implemented Project RED. This report characterizes contextual factors influencing their decision making process and motivations behind adaptations of the RED protocol and the impact of context and adaptations on implementation and sustainment of RED in these settings.

## Methods

### Hospital selection

Five hospitals in Northern California received two years of funding from a private foundation to implement RED. For their privacy, they are referred to as Hospitals A-E. The demographics and organizational characteristics of the participating hospitals are shown in Table 1. A team of researchers from Boston Medical Center where Project RED originated trained each hospital’s implementation team onsite for one day on implementing the RED toolkit (Additional file 1), and provided resources on how to deliver RED, how to monitor RED implementation and outcomes [11].

**Table 1:** Profile of participating hospitals

Hospital	Location	Hospital type & number of beds	RED implementation start and target pop:	Average # discharges annually (2009-2014)
A	Suburban/Urban	Military hospital. 205 beds. Fully implemented EMR.	Implemented in June 2012. 100% of patients in general medicine and surgery	4356
B	Suburban	Safety net, community, tertiary care, non-profit. 160 general acute care beds, 14 ICU. Fully implemented EMR.	Implemented in Nov 2012. 100% of patients 18 + in general medicine and surgery	(Acute care only, medical and surgical) 7967
C	Urban	Teaching/academic, safety net, community, non-profit. 375 total beds (180 acute care). Partially implemented EMR.	Implemented in 2013. Target pop originally ≥ age 55 for AMI, PNA, COPD patients; then ≥ 18 for patients with CHF, now all adults	16,905
D	Suburban	Safety net, community, non-profit. 217 beds. Fully implemented EMR.	Implemented in Nov 2012. Target patients at highest risk for readmissions.	(Inpatient only) 7856
E	Urban	Teaching/academic, community, non-profit. 313 beds. Fully implemented EMR.	Implemented in Sept 2013. All adults.	12,564

### Site visit data collection

A 5-member research team conducted site visits at all five hospitals implementing RED. All of these sites received Project RED training by the BMC RED research team prior to their implementation of RED. Site visits were conducted over a five month period between January and May of 2015. After obtaining written informed consent, we conducted one-on-one interviews and focus groups with 64 individuals, including hospital leadership and administrators, members of the RED implementation team, non-RED staff, and community-based ambulatory partners (Table 2). The interview guide is attached in Additional file 2. In addition, researchers shadowed RED providers at each hospital to assess the fidelity of the implementation of the RED components in the clinical setting including content of follow up phone calls, medication reconciliation procedures, appointment planning and transition of discharge plans to ambulatory providers. All hospitals were compensated $750 for their collaboration and contribution to the study. The Boston University School of Medicine/Boston Medical Center IRB approved this study protocol. The site visit objectives were:

- ▪ To gain insight and impressions on the experience of implementing RED, decisions regarding adaptation of RED components and the impact of these on RED sustainability.
- ▪ To directly observe the service delivery to see the discharge process in action and to learn from the staff involved in the implementation and delivery of RED.
- ▪ To construct a conceptual model of how contextual factors and adaptation strategies influence/hinder/support sustainable implementation.

**Table 2:** Breakdown of interviewed participants across all five participating hospitals

Participants	Number
Senior Leadership & Hospital Executives:	11
Clinical RED Implementation Team:	22
*Ex: Doctors, Nurses, Pharmacists*	
Non-Clinical RED Implementation Team:	19
*Ex: Social Workers, Transitions Coordinators, Data Analysts, Dieticians*	
Non-RED Staff:	9
Community Based Organization Partners:	3
TOTAL:	64

### Data analysis

Key informant and focus group interviews were audio recorded and transcribed verbatim. Two research assistants coded each transcript independently using NVivo [12], and all discrepancies were resolved through negotiation with a third party present. Each transcript underwent two rounds of coding, utilizing a modified grounded theory approach with constant comparative analysis. Codes were then collated into potential themes. Themes were refined and analyzed, and an overall framework for the data was developed.

From analysis of the five site visits, we constructed a conceptual model of contextual factors identified as influencing hospitals’ decisions to adapt the RED protocol and impacting whether it continued after external funding lapsed. We identified and characterized *external* and *internal* contextual factors influencing RED adaptation decisions, RED implementation experiences, and its sustainability. External factors are forces related to economy, government policy, and external financing or community level drivers. External contextual factors were generally immutable. Internal factors relate to hospital organizational structure and culture, leadership, and management. We defined adaptation of RED as an instrumental change to a RED component from the original RED protocol or eliminating one or more of the 12 RED components from the hospital’s planned program implementation. Using the framework defined in the U.S. Department of Health and Human Services’ State of the Art Review on fidelity and adaptation in substance abuse prevention, adaptations typically came in the form of additions (i.e. adding components to RED), deletions (i.e. deleting components) or modifications (i.e. maintaining components, but altering how they are done) [13].

**Fig. 1:**
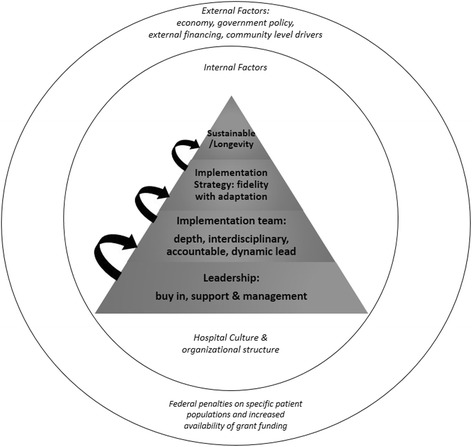
Schematic profile of the components needed for sustainable implementation of Project Re-Engineered Discharge (RED). All participating sites were given funding to implement Project RED at their hospital. Supportive, invested leadership (1), a multi-disciplinary, accountable implementation team (2), an appropriately adapted implementation strategy (3) and an empowering hospital culture (4) were all needed for RED to be sustainably integrated into hospital protocol and culture (5)

**Fig. 2:**
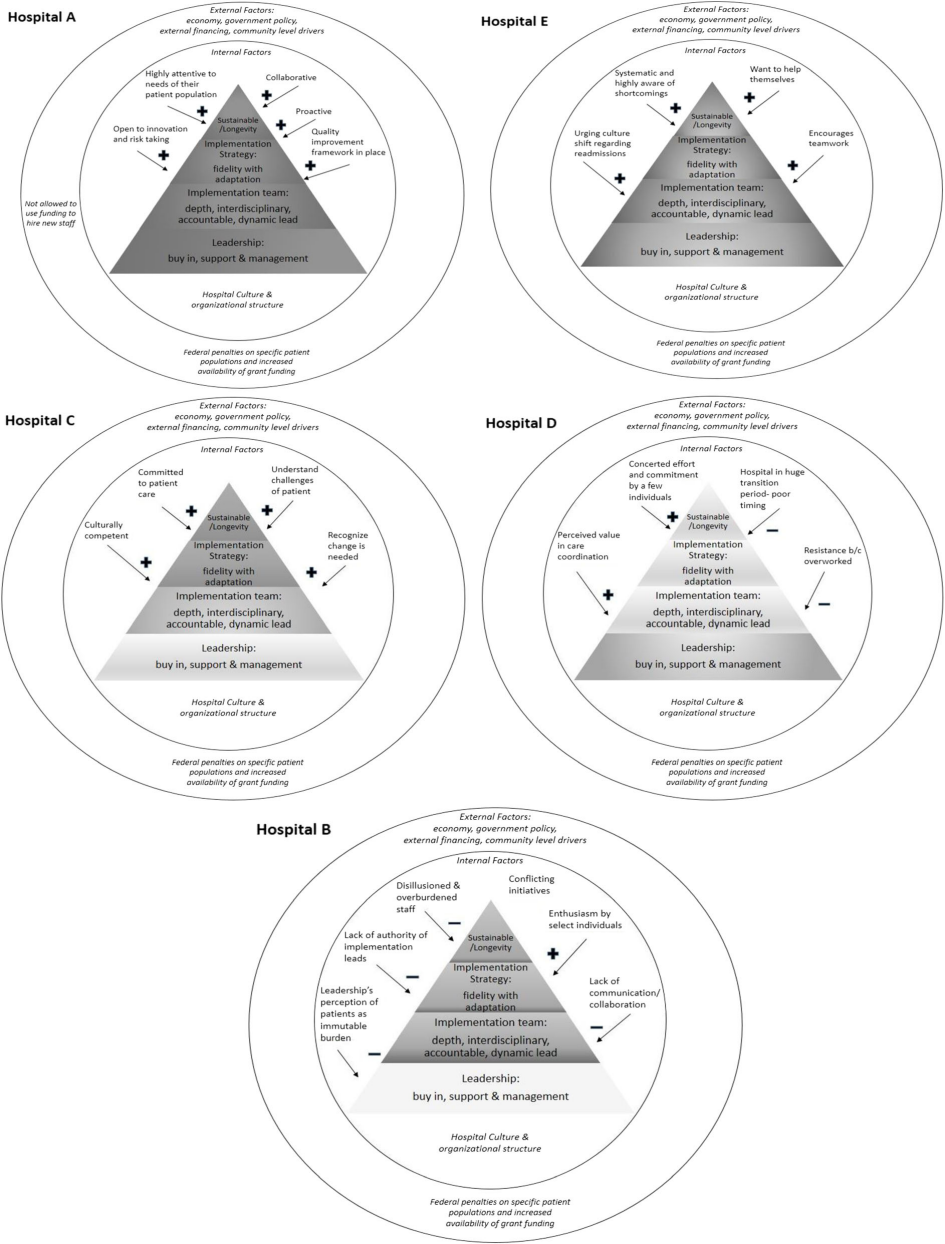
Site specific RED implementation schematics. Ordered from highest level of RED implementation success to lowest: Hospital **a**, **e**, **c**, **d**, **b**. Faded colors, as compared to the colors for Hospital A, indicate less success in those areas. Brighter colors indicate higher success components. Contextual influences of hospital culture are shown surrounding each pyramid

We characterized each hospital’s profile in terms of its organizational assets and deficits in each contextual domain. We used constant comparative analysis to identify criteria of the relative strength or weakness of each hospital in each contextual domain (see Table 3). An optimal context for sustainable implementation of Project RED is defined as a hospital environment that is strong in all internal contextual attributes and resilient or responsive toward identified external factors (Fig. 1). We created a unique implementation profile for each hospital (see Fig. 2).

**Table 3:** Strengths and concerns of each contextual factor

Contextual factor	Strength	Concern
RED as a Priority to Leadership	Leadership demonstrated buy-in by making RED an institutional priority. They also showed involvement, and support of RED implementation, and encouraged employees to embrace change, adaptation and creative solutions.	Leadership showed lack of focus on addressing readmissions and failure to commit adequate resources. There was also an absence of leadership involvement in RED implementation, and lack of guidance and direction from management.
Adaptation and Implementation strategy	Implementation strategy started with a purposeful planning period and careful deliberation on how to best implement RED. Adaptations maintained a high level of fidelity to the intention of the intervention.	Implementation strategy was unplanned, disorganized, and approached RED as a time-limited project. Focused on select elements of the RED toolkit, thereby failing to address critical aspects of the discharge process and inherently changing the possible impact of RED.
Implementation Team	Leadership selected an implementation team that had depth, was accountable, was multidisciplinary and had a dynamic leader who was able to effect change. Components of the RED toolkit were divided amongst enough individuals to delegate and distribute the workload, and where each person had a distinct role to play.	Implementation team lacked multidisciplinary input and representation; team often lacked the social capital and ability to influence others to be enthusiastic about RED implementation. Components of the RED were assigned in a manner that was burdensome to staff and lacked accountability.
Planning for Sustainability and Longevity	Forward-thinking planning to approach RED as a transformational process, rather than a project, with clear goals for integration into daily workflow.	Approached RED implementation as a grant-dependent project without consideration for sustainability of RED staff salary support or workflow integration of RED discharge process.
Hospital Culture	Positive hospital culture that embraced failures, fostered a feeling of empowerment for both employees and patients, and remained patient-centered. Leadership was supportive of implementation team, which promoted the feeling that chance was possible, fostering a spirit of continuous improvement.	Negative hospital culture that lead to employees holding defeatist attitudes towards their patient populations, felt helpless in effecting positive change in their environment, and failed to see discharge as a necessary area for improvement.

We defined *fidelity* as the degree to which a hospital implemented the 12 components of RED according to the RED Toolkit protocol. If the hospital implemented an adapted version of one or several of the RED components, we determined if the adaptation maintained or fundamentally changed the objective of the component as intended by the developers of RED. If the adaptation was responsive to hospital culture and context while maintaining the objective of the component, fidelity to the RED protocol was deemed high. If the adaptation substantially changed the objective of the RED component, fidelity was deemed low. For example, the objective of the 2-day post-discharge phone call, one of RED’s 12 components, is to assess for medication-related problems and take clinical action if a problem is identified. If a hospital employed a community health worker to complete the 2-day post discharge phone call, given the different skill sets of a community health worker versus a pharmacist, this would alter the potential efficacy of the phone call. Thus the objective of the component would be fundamentally changed, leading to low fidelity of this adaptation. We defined sustainability as the hospital’s ability to sustain the original or an adapted, site-specific version of RED for at least six months beyond the implementation period. Finally, we deemed hospitals to be successful when they were able to implement RED sustainably.

## Results

### External vs internal contextual factors

Participants identified both internal and external contextual factors influencing RED implementation and sustainability [14].

#### External factors

Two prominent external contextual factors were: 1) the focus of federal penalties on specific patient diagnostic populations (heart failure, pneumonia and heart attack) and 2) the increased availability of grant funding and technical assistance resources to incentivize US hospitals to improve care transitions. Both factors were largely attributable to peaked national interest in readmissions as a patient safety issue, public reporting of hospital readmission rates and the commencement of federal penalty policies.

For participating hospitals, the availability of external funding and the terms of the grant funding source were relevant in several ways. Firstly, the funder allowed the awardees the flexibility of choosing the transitional care program that best fit their organization. Secondly, grant funding incentivized implementation of care transition initiatives that hospitals may not have undertaken otherwise or may not have commenced at the particular time the funding was made available. The result was that hospitals varied in their organizational readiness. In one hospital, the concurrent availability of multiple funding opportunities resulted in simultaneous implementation of two care transition programs that led to staff confusion and lack of accountability for RED specific implementation outcomes. Thirdly, the foundation grant funds were earmarked for nursing care initiatives, but did not set parameters for awardees about how much nursing to use in the implementation of RED. However, in some cases it did promote a strong nursing focus for RED implementation. This focus turned out to be detrimental in Hospital D where the RED implementation was viewed exclusively as a nursing initiative rather than a shared, hospital-wide priority with the multi-disciplinary support necessary to succeed. Thirdly, the availability of external grant funding without a mandatory matching pledge of hospital resources limited the required engagement of hospital leaders in certain hospitals to ensure that RED implementation was an organizational priority with strong leadership commitment. This was best exemplified by the lack of accountability linked to the outcomes of the RED implementation effort, most noticeable when grant funding ended.

The public reporting of readmission rates only for specific patient populations, namely Congestive Heart Failure, pneumonia and Myocardial Infarction also influenced implementation. Public reporting of readmission rates on the Hospital Compare website served to increase interest among hospital leaders to address problems driving up readmissions. However, this incentive did not necessarily translate to a steadfast commitment specifically to the RED implementation effort but rather incentivized 3 participating hospitals to implement RED with only these targeted patients. This limited the responsibility of delivering RED to certain staff or hospital wards and often hindered teams from capturing organizational buy-in and cooperation needed. Conversely, the hospital that delivered RED to all high risk patients throughout the hospital reported better uptake and engagement among hospital staff to support the RED care team.

#### Internal factors

Internal or organizational contextual factors associated with effective adaptation and sustainability of RED were identified. These included: (1) RED as a *leadership priority with* buy in, involvement and support. (2) A dedicated multidisciplinary *implementation team* with expertise in quality improvement programs, depth, accountability and a dynamic implementation lead. (3) A well-planned *implementation and adaptation strategy* that maintained a high level of fidelity to RED protocol while adapting for organizational culture and resource availability. (4) Planning a *sustainable* integration of RED into the hospital’s future, and (5) Positive, supportive *hospital culture* focused on achieving better patient care. Figure 1 depicts the relationship between these five components and the external or macro level contextual factors.

### RED as a priority to leadership

Visible and committed leadership provided the foundation for a sustainable implementation of RED and resulted in adaptations to RED that fit organizational culture and operations. Participants defined effective leadership to be when leadership showed buy-in, involvement, and support of RED implementation and its sustainability. Senior leadership expressed buy-in by making RED an “institutional priority”, showing commitment to its mission and purpose even when there were challenges or when supplemental funding ran out:

“*We would not apply…for a program that didn’t align with one of our priorities… we had to be committed to…do it, even if we weren’t funded … we would have to be committed to keep it going.”*

When hospital leadership did not believe RED was a priority, it showed in their lack of focus on addressing readmissions and failure to commit adequate resources to the effort:

*“We had [a] previous administration and then [the] new administration came in and their main focus was that the front doors of this hospital will shut down if we don’t fix the financial issues. So, the quickest and easiest way to do that? Lay off a bunch of people… there’s a big struggle between… staffing and finding the money to do [Project RED].”*

After initial buy-in, effective leadership then needed to support and provide managerial guidance throughout RED implementation. A senior leader from a successful hospital explained,

*“[We, the leadership] get into the workplace where the work’s being done … we go see what’s actually going on so that the decisions that we make at a higher level, from that 30,000 foot level, aren’t just these sweeping changes that people are like, ‘Woah, what are you doing?’ We don’t guess. We actually know.”*

Leadership at this hospital was also “supportive of… crazy ideas that… ultimately are really good ideas,” encouraging employees to embrace change, adaptation and creative solutions:

*“When you have leadership at the very top that… is supporting, is rewarding, is acknowledging people… for taking risks. And saying, “A failure is not necessarily bad. It’s just an opportunity to learn.”…I think that creates an environment that people are willing to step outside of their comfort zone to serve a common good and to serve a common mission.”*

When leadership was unsupportive, this usually presented itself as an absence of leadership involvement in RED, and a lack of guidance, resources and direction from management. A staff member from a hospital that was unsuccessful in sustainably implementing RED expressed,

*“Without…direction from management saying, ‘It’s this way. Everybody will do it. This comes first, that comes second’… [Project RED] is not a key, central component of the system… the executive team needs to provide firm direction as to what’s the workflow, what are we trying to accomplish, what are the expectations, and then hold people accountable for whatever that is.”*

Hospitals that lacked effective, present and visible leadership struggled to implement RED.

### Adaptation and implementation strategy

We learned that adaption of Project RED is necessary for a sustainable implementation but adaptations must maintain a high level of fidelity. Examples of addition type adaptations include adding the discussion of advanced directives by the discharge educator at Hospital D as part of the RED protocol and adding a component from the Care Transition Model. Deletion adaptations were common and examples included elimination of the component to require availability of the discharge summary within 24 h of discharge, and the confirmation of a post-discharge follow up appointment. Alteration type adaptations included substituting a community health worker for the pharmacist to complete the post-discharge follow up call. Not all adaptations were effective. In some cases their success was influenced by the organizational context.

Successful implementation involved a well thought-out plan of action, adherence to all elements of the RED toolkit, site-specific adaptation, and a monitoring and quality improvement framework to guide implementation. Weak implementations were unplanned, disorganized, and approached RED as a time-limited project, rather than a quality improvement initiative integrated into the future of the hospital.

The most effective implementation strategies started with a purposeful planning period and careful deliberation on how to best implement RED in the specific hospital. The hospital with the most successful RED implementation took a full year to, “*vet through all the different…initiatives,”* selecting, *“Project RED as a group, because it fit our model*.” They took another year to fully plan the implementation, completing a root-cause analysis to identify reasons for readmissions at their hospital and identification of high risk populations. Hospitals who were unsuccessful in implementing RED skipped this planning period and root-cause analysis, and rather proceeded rapidly, without a clear implementation strategy and with considerable trial and error.

Maintaining high fidelity to the 12 steps of the RED toolkit was crucial for a sustainable implementation. An implementation lead at a highly successful RED hospital explained that when introduced to the RED toolkit she was told:

*“This is your bible. Don’t deviate from it. You can add to it, but as long as you do these core things, you can have latitude to do whatever you want to do’… you can’t do Project RED and do element nine and five and four…You gotta do all twelve elements. You can’t pick and choose.”*

Unsuccessful implementation strategies focused on select elements of the original RED toolkit, thereby failing to address critical aspects of the discharge process. This suggests that the way to comprehensively address the discharge process, was to give attention to each of the 12 RED elements thoughtfully and fully.

That said, adapting the RED toolkit site-specifically was also essential in many cases. One hospital that initially struggled to implement RED, later found more success when they adapted their implementation strategy:

*“We tried to mirror Boston… but we realized… every hospital’s unique …. [after that] they came up with the transitional care nursing program. And the beauty of it is that we’ve evolved… and if it doesn’t work, we’re like, ‘Ok. Well, we gotta fix it. We gotta change it.’”*

At the aforementioned successful hospital, despite describing the RED toolkit as their ‘bible’, they also made additions. Most notably, this hospital added technology to enhance RED efforts; although it took a while to work out the flaws, all pharmacists now have mobile devices with a medication reconciliation application on them to help implement element five, and at the time of the site visit, they were looking to add ‘Louise’, the RED virtual patient advocate, to help with teach back for elements 7, 8 and 9. This hospital was also the only site to add a ‘patient advisor’ to the implementation team, a member of the patient population who spoke for and represented the patient perspective. This showed a crucial adaptation to their RED strategy, as it allowed their approach to reflect the needs of the local patient population. These adaptations were beneficial because RED had a solid working framework and presence at this hospital. By contrast, in hospitals that never established a RED framework, additions to the original toolkit proved distracting. For example, the hospital with the lowest success added elements of Project Boost and the Coleman Care Transitions Program to RED implementation, changing significantly RED method. These additions, however, failed to produce any concrete results.

Rather than add components to RED, some hospitals decided to adapt RED by deleting parts of the original toolkit. At a hospital that implemented RED sustainably, they chose to forgo use of the after-hospital care plan (AHCP) because they were already giving patients a chart of their medications at discharge, and had separately developed a standard discharge instruction template for employees to use as a RED checklist. They believed adding an additional document would be repetitive and burdensome, and thus eliminated it. Because they addressed aspects of the AHCP elsewhere, this deletion did not detract from RED success, and rather saved potential time and confusion.

Many hospitals deleted one or more of the twelve RED elements, inherently changing the possible impact of RED. Typically elements were dropped if resources or personnel were limited. For example, one hospital did not address element 4 pertaining to post-discharge outpatient services and equipment because they did not have any home health services, and had “oversaturated” their community based organization partners. Several others skipped element 6, reconciling the discharge plan with national guidelines, but the reasons for these decisions were not given. In fact, only two hospitals addressed all 12 elements; they were also the two most successful hospitals in sustainably implementing RED, suggesting that adapting RED by deleting some of its core elements may not lead to optimal RED results.

Adaptation in the form of alterations or modifications was also common, and resulted in varied success. Several hospitals experimented with the qualifications of personnel used to fill the role of the ‘discharge educator’ and to administer follow up phone calls. In the clinical trials of RED at BMC, the discharge educators were nurses who completed all RED tasks except the follow-up phone call which was done by pharmacists. In three of the five implementing hospitals, nurses fulfilled the discharge educator role, but in the two remaining hospitals patient care advocates and social workers took these responsibilities, changing, by nature of the skillset of the discharge educator role. The hospital that used social workers renamed the role of ‘discharge educator’ to ‘discharge coach’ as they recognized the social workers were not in charge of delivering any health education; rather they were present in the room when nurses and doctors talked to patients to provide support. This hospital, however, is phasing out social workers from their team, using nurses instead, given, *“[Social workers] just don’t have the transformational… skill-set to engage the rest of the interdisciplinary team.”*

Many hospitals had non-pharmacists place follow-up calls, opting instead for nurses or patient care advocates. In the hospital where the patient care advocates made the calls, the content was more social than clinical, something the original RED toolkit advised against. Luckily, however, pharmacists also made a 2-day follow up call at this hospital, covering the clinical aspects of after-hospital care. In hospitals where nurses made the call, this substitution did not seem to negatively affect the after-hospital clinical care of the patient, as nurses were still medical professionals, and often coordinated closely with pharmacists. One hospital found the follow up calls with nurses highly successful once they made a slight modification. Their follow up calls originally were conducted by nurses who had not visited the bedside, but patients did not respond well to this, questioning, *“Who are you? Why are you calling me? I don’t know who you are”*. When this hospital switched to have the same bedside nurse also make the follow up phone calls, this hospital was better able to retain their patients and their patients were, *“more likely to be honest in talking about what’s going on with hospitalization…when they know who the person is, when they have that rapport and that trust.”* This adaptation helped this hospital improving their discharge and follow-up care, and might not have been possible if they used pharmacists in this role.

Included in the Project RED toolkit [11] are guidelines on how to monitor implementation and outcomes of RED. However, not all sites opted to track their implementation efforts. Hospitals that implanted RED sustainably, had clear ways to monitor progress, using data collection and analysis of their readmissions, deciphering which readmissions might have been preventable, and holding weekly or monthly meetings to discuss the progress of RED and its effect on readmissions and the discharge experience. Hospitals that were able to sustain RED implementation, lacked data monitoring systems and accountability on individual and organization levels, leading to uncertainty as to whether RED implementation had reduced readmissions.

Having a quality improvement framework already in place was beneficial for implementation, as it gave structure and familiarity to implementing a new project, and encouraged ongoing adaptation and monitoring. Top performing hospitals utilized Lean methodology [14] as well as the principles of PDSA (plan-do-study-act), both of which encouraged making small changes as needed and continuous evaluation of these processes. Hospitals that did not implement RED sustainably, typically did not use these methods, contributing to unsystematic efforts.

While it did not seem essential for sustainable RED implementation, technology was commonly used to aid RED implementation. For hospitals whose implementation efforts were highly or moderately sustainable, technology enhanced RED efforts. For example, at one hospital, software named “Meducation” which translates discharge instructions into multiple languages as well as for individuals with low-literacy, aided the pre-existing efforts to address the language component of the RED toolkit. Conversely, when technology was adopted in hospitals who were struggling to implement RED, it often made a bad situation worse. This was the case in the hospital with the lowest level of RED success:

*“The entire nursing application had to be…redesigned and rebuilt … but then we couldn’t implement [RED] because they had to retrain everybody on how to use the [new] clinical application. So we had a 2-3 month gap [due to] technology delays.”*

When RED eventually did deploy, resistance had developed towards adding more technology. RED team members admitted: *“Frankly it was just overload. It was too much for the staff, it was too stressful… [There was] burn out on technology already before we even launched [RED].”* The imposed technology created new problems that masked the existing problems in hospital leadership, teamwork and implementation strategy.

### Implementation team

The implementation team was a key component of sustainable implementation of RED. In hospitals with positive RED implementation experiences, senior leadership selected an implementation team that had depth, was accountable, was multidisciplinary and had a dynamic leader who was able to affect change. Implementation teams were most successful when they involved enough people to make a noticeable difference. When teams were only two or three people, they usually failed to reach a large enough number of patients to see RED have a measurable effect on readmission rates, and also did not have the capacity to address all 12 components of RED.

*“We make a very positive impact on the patients that we do see, but when we’re down-staffed to such degree, we see a very small number of patients… so we’re not actually impacting the overall readmission rate for the hospital.”*

At the same time, too large a team was equally problematic by leading to lack of accountability. At one hospital that struggled with RED implementation, they chose to “train everyone on RED.” One employee there believed this lack of delegation and individual responsibility,

“*makes [Project RED] nobody’s job, ‘cause when it’s everybody’s job, it’s nobody’s job… we did not inherit a hospital that has a culture of accountability…we get to pick and choose… We don’t even know if Project RED is or is not an effective intervention in our environment because we’ve never had all of the pieces in place for any period of time consistently.”*

The most effective teams were those where the components of the RED toolkit were divided amongst enough individuals to distribute the workload, and with each person making a distinct contribution. A highly successful hospital *“assigned… champions to every [element]”,* explaining that*, “the key is having somebody take ownership of it [each element] and having a manager … to audit it and keep it in line”*. The team implemented all RED toolkit components, while ensuring that individual element champions worked together towards the ultimate goal of reduced hospital readmissions.

The most successful implementation teams were multi-disciplinary, including all the professions involved in discharge and care transitions: doctors, nurses, social workers, case managers, administrators, home health representatives and pharmacists.

*“It’s gotta be multidisciplinary to eliminate the perceived barriers… you have to have the right people at the table and it can’t just be nursing and it can’t just be medicine.”*

When implementation teams were comprised of only one discipline, for example only nurses or only administrators, hospitals found it difficult to engage all the people required to change the discharge process. One hospital that had significant challenges implementing RED, and had an implementation team consisting of just two administrators described:

*“To get people to collaborate and for the good of the whole [is challenging] …Physicians are generally pretty autonomously functioning. They don’t like to be told what to do… Especially with regards to… readmission.”*

Even when team members were well-intentioned and motivated to make RED sustainable, they often lacked the social capital and ability to influence others to be equally enthusiastic about this endeavor. In part this came from lack of senior leadership support, but part stemmed from individuals simply not being the “right fit” for the job, as they lacked assertiveness or decision making authority. By contrast, highly successful implementation teams had one or more dynamic leaders who were able to galvanize others to make changes and work towards reduced readmissions. The leadership from a hospital that implemented RED sustainably explained:

*“One of the things that… [we] do when we build the team, anything that touches Project RED, is… make sure we have the right fit for the right position… the element champions… they’re go-getters … they get stuff done.”*

### Planning for sustainability and longevity

RED was sustainable past the end of grant funding and enculturated in the hospital’s mission only when the leadership, implementation team and implementation strategy were effective. Sustainability and longevity also required forward-thinking and planning to approach RED as a process, rather than as a project. Two hospitals with the highest success of implementing RED talk about this approach:

*“Don’t start this [RED] off as a project. Start this off as a process that you are going to adhere to forever. You’re going to continue to modify it and improve it, PDSA [plan-do-study-act] it… The standardization of the discharge process is more important than chasing that outcome [lower readmission rates].”*

*“My goal with Project RED is to have a focused team looking at this process – the twelve criteria … [so that] at the end of this two-year program … this becomes just standard work for all case managers, social workers, bedside nurses, and doctors. So that it’s enculturated.”*

The true mark that RED had achieved visibility and sustainability was when employees not involved in RED not only knew of it, but also began to regard those working on RED as “experts” or “consultants” on readmissions, and requested their expertise to help improve areas not originally in RED’s scope of influence or responsibilities.

By contrast, hospitals that approached RED as a grant-dependent project failed to achieve prolonged influence, and failed to continue RED efforts at a similar level of intensity both during the funding period and thereafter. The hospitals with the lowest success of RED implementation typically perceived lack of funding as the primary reason for lack of sustainability, when in most cases, it was due to a breakdown in leadership, implementation teamwork, and poor implementation preparation. Hospitals often used their funding to hire personnel (administrators, care-transitions coordinators, data analysts). While this may have been effective in the short-term, once funding ran out these hospitals either terminated these individuals, or modified their roles away from RED-specific responsibilities. Hiring personnel specifically to support RED efforts was not the main issue, the problem arises when this new personnel is supported only by grant funds and there isn’t a plan in place to keep them once funding runs out. This can be avoided by using existing staff, already embedded in the hospital’s budget to deliver RED. Hospitals that sustained RED after the end of funding, used their grants on equipment and technology, and to train existing employees for RED.

Unsustainable integration of RED responsibilities into other roles was a struggle at all but one hospital. An employee at a marginally successful RED hospital demonstrated this, admitting:

*“The term Project RED is really kind of getting smaller here…the organization has changed… if you asked a nurse here that’s been here less than a year, ‘Tell me about Project RED, ’ they would not know what it is.”*

### Hospital culture

Hospital culture either positively or negatively permeated all implementation efforts, and subtly influenced the way hospital personnel approached RED. Positive hospital culture was one that embraced change and failures, fostered a feeling of empowerment for both employees and patients, and remained patient-centered during successes and setbacks. This often corresponded with effective leadership who, by supporting their implementation teams, created the feeling of, “*empowerment of people at the front lines to affect the environment with which they live and work.”* Believing change was possible fostered a spirit of continuous improvement in successful hospitals, urging employees to go the extra mile for their patients;

“*[We] just are very fortune…to have a group of people that are really dedicated to giving really good patient care… and to doing the right thing…we’ve really started a culture of improvement and continuous improvement… Bring [an idea or issue] to this meeting and it gets done…I don’t think we’ve had a single thing yet where somebody’s like “No, we can’t do that” … they’re like “Oh, yeah, no problem. We can do that.” … so definitely hospital culture plays a role in feeling … like you have this professional agency. There’s a good chance that you can do something about it.”*

By contrast, negative hospital culture was observed when employees held defeatist attitudes towards their patient populations, felt helpless in effecting positive change in their environment, and failed to see discharge as a necessary area for improvement. In one hospital with very low implementation success of RED, hospital employees expressed feeling like they were being “dumped on” with undesirable patients like “chronic inebriates” and elderly patients who experience high rates of readmissions. These attitudes lent themselves to a pessimistic view of RED as a viable solution for these patients:

*“There’s chemical dependency, alcohol related stuff, there’s chronic abdominal pain, chronic pancreatitis… those people are probably gonna need something different than just Project RED. It ain’t gonna be enough.”*

This played into a feeling of hopelessness that RED, or any other quality improvement measure, could actually improve hospital problems:

*“Everybody has their thing that they identify as problems and a lot of us just live with it.”*

## Discussion

Implementation of quality improvement measures, in particular programs involving care transitions, are often difficult to translate from theory to practice. Even harder, is for these quality improvement initiatives to become so successful, that they create lasting change in hospital functioning and outcomes [15–18]. Given the different institutional priorities, leadership, personnel and culture present at each site, the five recipient hospitals approached the task of implementing RED at their locations very differently. Amongst sites that sustainably integrated RED, we found commonalities in five key areas: effective leadership, implementation teams and implementation strategies, supportive culture, and attention to developing sustainable, long-term plans.

For each of these five components, there is ample literature to support their importance. Our findings are highly consistent with those we found in studying ten RED implementation sites across the country in 2011. From that study, it was similarly found that active leadership, a multi-disciplinary team, a dynamic implementation lead, fidelity to the RED toolkit and site-specific root cause analysis were all necessary for sustainable RED implementation [10]. Other studies exploring how to implement evidence-based clinical practices and care transition interventions similarly confirm the importance of leadership support and commitment, as well as a supportive organizational culture with the urgency to improve [19–22].

What our current study suggests is that these components may be dependent on each other, and can be arranged in hierarchical order as depicted in Fig. 1. We advance that without leadership (the base of the pyramid) and positively reinforcing hospital culture (the context surrounding implementation), it is difficult to have a strong implementation team, implementation strategy or sustainability of RED. The vital importance and influencing power of leadership and positive culture are well explored and supported in studies looking at person-organization fit. It has been found that employees feel more committed and will stay longer at organizations whose ethical values and missions align with their own, suggesting that promoting positive hospital culture that embraces change and strives towards improved patient care, may attract employees with these same priorities [23]. In the context of RED, this implies that a strong implementation team may result from a strong hospital culture.

Perhaps the ultimate goal of any quality improvement measure is sustainability, and therefore we place it at the pinnacle of the successful implementation scheme (Fig. 1). In the experience we report, although all other components needed to be in place before sustainability could be achieved (effective leadership, implementation team, strategy and hospital culture), sustainability truly needed to be part of the initial vision and plan for RED, even before implementation efforts began. The literature supports that in order for quality improvement initiatives to be effective, hospitals must attempt to fully integrate them into everyday routine and standard hospital protocol, rather than approach them as isolated, time-bound projects [10, 19, 20, 22, 24, 25]. Only hospitals that approached RED as a ‘process’ rather than a ‘project’ were truly successful in sustainably implementing RED, and making lasting changes in streamlining their discharge procedures. The original RED toolkit does not give preference to incorporating RED using existing staff, however, from this study we learned that integrating RED into already existing systems and staff was an important strategy for success. This is an important addition that could be made to the RED toolkit.

Hospitals that maintained fidelity to RED tended to express higher satisfaction with their implementation experience. However, adaptations to RED were common, and their impact was variable. Modifications seemed more sustainable when implemented within the framework of a quality improvement model, whether it be the Lean model, PDSA, or a handful of other frameworks [26]. These frameworks not only gave structure and direction to making changes to RED, but also ensured monitoring of those changes to provide objective measures of overall improvement. Adaptations also tended to aid implementation efforts when made with the purpose of enhancing effectiveness and process improvement, rather than changes made due to financial, time or staff restraints. Literature in implementation science advances that while adaptations are inevitable, there is often a tension between fidelity and adaptation, as we found in this study. Adaptation can improve implementation and sustainability [27], but the way to best go about modifying interventions remains unclear. Most studies advise understanding the theory behind an intervention, and distilling out its core components before modifying its content, somewhat like identifying the main ingredients of a recipe that cannot be substituted or changed [13, 28]. The balancing act between fidelity and adaption in harmony rather than contrast, however, is still little understood and warrants further study.

Ultimately, RED may not have been the right fit intervention at all sites; this could have been because of inopportune timing, for instance when hospital leadership was changing and staff was being terminated, or because RED was implemented by the people who were unsupportive or lacked the leadership power to galvanize change. The implications of this may be that certain hospitals lack a sound springboard for RED implementation, and there may be ‘pre-requisites’, including (1) leadership seeing RED as an institutional priority, (2) a vision to incorporate RED sustainably into the hospitals future and (3) a positive, empowering hospital culture. Even before funding is given to hospitals to implement RED, it may be advisable for them to assess if they possess these qualities.

Some limitations must be noted. Site visits were conducted at varying times following completion of implementation and it may have affected our ability to observe the implemented version of RED in the hospitals that were no longer doing RED. In those two hospitals where we were not able to observe the implementation of RED, we held key informant interviews and focus groups with those involved in RED when it was implemented. During site visits, the hospitals selected which personnel would be interviewed by our research team, which may have skewed our impressions either positively or negatively if their perspectives were not representative of the majority of employees. It is likely that a degree of social desirability response bias occurred, however this was mitigated by attempting to speak to a wide array of individuals involved in RED in different roles and capacities [29]. The short nature of the site visits may not have been enough time for the hospitals to adequately showcase RED operations at their site, as not all desired interviews with hospital leadership and administrators, members of the RED implementation team, non-RED clinical staff, and community-based ambulatory partners took place at each location.

Additionally, deciphering RED’s impact on hospital readmissions poses some challenges. Most hospitals did not deliver RED to all patients, but rather chose specific high-risk groups to receive the intervention. When recording readmission statistics, however, these hospitals did not keep track of rates specific to these specific groups, and rather just overall readmission rates for the hospital. This may have masked the impact RED had on readmissions. Alongside RED, most hospitals were simultaneously implementing other quality improvement projects involving similar initiatives such as increasing communication between providers, and partnering with community based organizations to improve care post-discharge. The effects of these initiatives cannot be separated from the impact of RED using our qualitative methods, thus factors beyond our scope of study may have also influenced changing readmission rates. For this reason, our study focused on the qualitative experiences and perceived impacts of RED implementation, rather than report on readmissions statistics, as it was impossible to distinguish RED from other factors influencing readmissions rates, and this data was inconsistently measured across the five sites. An additional limitation is that we studied only five hospitals, of which only three were successful. All hospitals were based in California and implemented RED during a limited range of historical time. This reduction in variability of the external environment, while helpful from one perspective, may reduce the generalizability of results to other states and time.

## Conclusion

The five components for sustainable RED implementation outlined in this study are pertinent to consider when implementing any quality improvement or care transitions initiative. More specifically, this paper offers insight and guidance for hospitals or other healthcare facilities who are considering implementation of RED. The importance of hospital culture, leadership and planning, including for sustainability, should not be underestimated in their influence on the success of RED and the efforts to reduce hospital readmissions.

## Additional files

Additional file 1: The 12 components of the RED Toolkit. (DOCX 13 kb)

Additional file 2: Interview guides for site visits. (DOCX 13 kb)

## Abbreviations

AHCP: After-hospital care plan
CCTP: Community-based care transitions program
PDSA: Plan-do-study-act
QIO: Quality improvement organizations program
RED: Re-engineered discharge
