# Supplementary material for: Implementation and adaptation of the Re-Engineered Discharge (RED) in five California hospitals: a qualitative research study
Source: BMC Health Serv Res. 2017 Apr 19;17:291. doi: 10.1186/s12913-017-2242-z (PMC5397802; doi:10.1186/s12913-017-2242-z)
Supplement: Supplementary file 1 — The 12 components of the RED Toolkit. (DOCX 13 kb) [file 12913_2017_2242_MOESM1_ESM.docx]

**Additional file 1. The 12 components of the RED Toolkit**

| **RED Component** | **Responsibilities** |
| --- | --- |
| **1. Ascertain need for and obtain language assistance.** | • Find out about preferred languages for oral communication and written materials.  • Determine patient and caregivers’ English proficiency  • Arrange for language assistance as needed, including translation of written materials. |
| **2. Make appointments for follow-up medical appointments and post discharge tests/labs.** | • Determine primary care and specialty follow-up needs.  • Find a primary care provider (if patient does not have one) based on patient preferences: gender, location, specialty, health plan participation, etc.  • Determine need for scheduling future tests.  • Make appointments with input from the patient regarding the best time and date for the appointments.  • Instruct patient in any preparation required for future tests and confirm understanding.  • Discuss importance of clinician appointments and labs/tests.  • Inquire about traditional healers and assure that traditional healing and conventional medicine are complementary.  • Confirm that the patient knows where to go and has a plan about how to get to appointments; review transportation options and address other barriers to keeping appointments (e.g., lack of day care for children). |
| **3. Plan for the follow-up of results from lab tests or studies that are pending at discharge.** | • Identify the lab work and tests with pending results.  • Discuss who will be reviewing the results, and when and how the patient will receive this information. |
| **4. Organize post-discharge outpatient services and medical equipment.** | • Collaborate with the case manager to ensure that durable medical equipment is obtained.  • Document all contact information for medical equipment companies and at-home services in the AHCP.  • Assess social support available at home.  • Collaborate with the medical team and case managers to arrange necessary at-home services. |
| **5. Identify the correct medicines and a plan for the patient to obtain and take them.** | • Review all medicine lists with patient, including, when possible, the inpatient medicine list, the outpatient medicine list, the outpatient pharmacy list, and what the patient reports taking.  • Ascertain what vitamins, herbal medicines, or other dietary supplements the patient takes.  • Explain what medicines to take, emphasizing any changes in the regimen.  • Review each medicine’s purpose, how to take each medicine correctly, and important side effects.  • Ensure a realistic plan for obtaining medicines is in place.  • Assess patient’s concerns about medicine plan. |
| **6. Reconcile the discharge plan with national guidelines.** | • Compare the treatment plan with National Guidelines Clearinghouse recommendations for patient’s diagnosis and alert the medical team of discrepancies. |
| **7. Teach a written discharge plan the patient can understand.** | • Create an AHCP, the easy-to-understand discharge plan sent home with patient.  • Review and orient patient to all aspects of AHCP.  • Encourage patients to ask. |
| **8. Educate the patient about his or her diagnosis.** | • Research the patient’s medical history and current condition.  • Communicate with the inpatient team regarding ongoing plans for discharge.  • Meet with the patient, family, and/or other caregivers to provide education and to begin discharge preparation. |
| **9. Assess the degree of the patient’s understanding of the discharge plan.** | • Ask patients to explain in their own words the details of the plan (the teach-back technique).  • May require contacting family members and/or other caregivers who will share in the care-giving responsibilities. |
| **10. Review with the patient what to do if a problem arises.** | • Instruct on a specific plan of how to contact the primary care provider (PCP) by providing contact numbers, including evenings and weekends.  • Instruct on what constitutes an emergency and what to do in cases of emergency. |
| **11. Expedite transmission of the discharge summary to clinicians accepting care of the patient.** | • Deliver discharge summary and AHCP to clinicians (e.g., PCP, visiting nurses) within 24 hours of discharge. |
| **12. Provide telephone reinforcement of the Discharge Plan.** | • Call the patient within 3 days of discharge to reinforce the discharge plan and help with problem-solving.  • Staff DE Help Line. Answer phone calls from patients, family, and/or other caregivers with questions about the AHCP, hospitalization, and follow-up plan in order to help patient transition from hospital care to outpatient care setting. |
