# Supplementary material for: Implementation and adaptation of the Re-Engineered Discharge (RED) in five California hospitals: a qualitative research study
Source: BMC Health Serv Res. 2017 Apr 19;17:291. doi: 10.1186/s12913-017-2242-z (PMC5397802; doi:10.1186/s12913-017-2242-z)
Supplement: Supplementary file 2 — Interview guides for site visits. (DOCX 13 kb) [file 12913_2017_2242_MOESM2_ESM.docx]

**Additional file 2. Interview Guides for Site Visits**

| A. Hospital Administrators/Leadership |
| --- |
| - How did your hospital decide to prioritize readmissions reduction? - How did you decide to implement Project RED? - What are the facilitators and barriers to the implementation of the RED? - Has the implementation of the RED had any effects on the hospital’s functioning? If so, how? - How did you obtain hospital staff buy-in? - How would you describe the culture of your hospital, and how does it support or challenge the discharge process/RED implementation? - How has the hospital measured progress of the RED implementation? |
| B. RED Implementation Team |
| - How has the RED been implemented? Did you modify Project RED for implementation at your site? How closely does the implementation align with steps in the RED toolkit? - What has been your experience with the implementation of the RED? (Facilitators/challenges) - Who are members of the RED team? What are their backgrounds? Were any staff members hired specifically for RED? - How are components in the RED toolkit assigned? I.e. who does what, and why? - How was the implementation team trained? - How have the team dynamics affected the implementation of RED services? - How do you identify your target patients? - Please describe your discharge process map and after hospital care plan. |
| C. Non-RED Clinical Staff |
| - What has been your experience with the implementation of Project RED? - Has the implementation of Project RED had any effects on your work in the hospital or hospital functioning? If so, how? - Do you have any suggestions on how the RED could be improved? - In your opinion, what has been the patient and caregiver experience with Project RED? |
| D. Community-based Ambulatory Partners |
| - What has been your experience with the implementation of Project RED? - Has the implementation of the RED had any effects on your work? If so, how? - Do you have any suggestions on how you could work more efficiently with the RED implementation team? - Is there any way you could contribute to the implementation of Project RED? |
